# You Are What You Eat: Food Limitation Affects Reproductive Fitness in a Sexually Cannibalistic Praying Mantid

**DOI:** 10.1371/journal.pone.0078164

**Published:** 2013-10-10

**Authors:** Katherine L. Barry

**Affiliations:** Department of Biological Sciences, Macquarie University, Macquarie Park, New South Wales, Australia; Hungarian Academy of Sciences, Hungary

## Abstract

Resource limitation during the juvenile stages frequently results in developmental delays and reduced size at maturity, and dietary restriction during adulthood can affect longevity and reproductive output. Variation in food intake can also result in alteration to the normal pattern of resource allocation among body parts or life-history stages. My primary aim in this study was to determine how varying juvenile and/or adult feeding regimes affect particular female and male traits in the sexually cannibalistic praying mantid *Pseudomantis albofimbriata*. Praying mantids are sit-and-wait predators whose resource intake can vary dramatically depending on environmental conditions within and across seasons, making them useful for studying the effects of feeding regime on various facets of reproductive fitness. In this study, there was a significant trend/difference in development and morphology for males and females as a result of juvenile feeding treatment, however, its effect on the fitness components measured for males was much greater than on those measured for females. Food-limited males were less likely to find a female during field enclosure experiments and smaller males were slower at finding a female in field-based experiments, providing some of the first empirical evidence of a large male size advantage for scrambling males. Only adult food limitation affected female fecundity, and the ability of a female to chemically attract males was also most notably affected by adult feeding regime (although juvenile food limitation did play a role). Furthermore, the significant difference/trend in all male traits and the lack of difference in male trait ratios between treatments suggests a proportional distribution of resources and, therefore, no trait conservation by food-limited males. This study provides evidence that males and females are under different selective pressures with respect to resource acquisition and is also one of very few to show an effect of juvenile food quantity on adult reproductive fitness in a hemimetabolous insect.

## Introduction

Nutrition is of paramount importance in organismal development, and marginal resources will impose severe constraints on individuals in most natural populations. Resource limitation during the juvenile stages frequently results in developmental delays and reduced size at maturity [1-4], and may also decrease the reproductive output of animals that produce gametes before adulthood or store juvenile resources for use during adulthood [5-7]. Dietary restriction during adulthood can affect fitness in a variety of ways, the most obvious being reduced adult longevity and reproductive output [8-10]. 

Variation in food intake can also result in significant alteration to the normal pattern of resource allocation among various body parts or life-history stages [11]. This change in the allocation of resources may be a non-adaptive response, where the finite pool of resources is always proportionally allocated to all traits irrespective of absolute intake (‘proportional resource allocation’), or an adaptive response involving the strategic allocation of resources to important traits (‘strategic resource allocation’). For example, some animals may preferentially allocate resources to functionally significant traits by sacrificing the development of other characteristics that are less important [12]. Short-lived caddisflies *Odontocerum albicorne* with fewer resources available at metamorphosis have smaller wings and thoraces as adults, but abdomen size is maintained irrespective of resource availability [13]. Conversely, in the long-lived caddisfly *Glyhotaelius pellucidus*, fewer resources at metamorphosis mean smaller wings and abdomen, but thorax size is maintained [14]. This variation is likely to be the result of different life history patterns i.e. short-lived species invest in the abdomen and therefore reproductive investment, and long-lived species invest in the thorax and therefore longevity.

 Praying mantids are sit-and-wait predators whose resource intake can vary dramatically depending on environmental conditions (e.g. temperature, rainfall, population density) within and across seasons [15,16], making them useful for studying the effects of juvenile and adult feeding regime on various facets of reproductive fitness. The significant skew towards the study of holometabolous insects in the resource allocation literature [11] further increases the appeal of studying these hemimetabolous insects. Previous studies of praying mantids have suggested that food limitation during the juvenile stages has an effect on development time and body size at maturity [15,17]. In addition, food limitation during adulthood is expected to have a significant negative impact on both body condition and gamete number, particularly in females [18,19]. That females use sexual cannibalism as a foraging strategy to increase fecundity [20,21] and are more likely to cannibalise when resources are scarce [18,20,21], provides further evidence that resource limitation is important in these insects. Male mantids are polygynous scramble competitors, selected for their ability to locate mates effectively [22,23] and perform in post-copulatory sperm competition [24,25]. Food limitation might, therefore, have a negative impact on various aspects of sperm production or on the size/body condition of males and their subsequent ability to locate potential mates or to continue mate searching over time. A lack of resources may also affect other functionally significant characters used in mate location and mate searching, such as antennae for detection of chemical signals [22,25-27] and/or wings for locomotion towards those signals. 

 My primary aim in this study was to determine how varying juvenile and/or adult feeding regimes affect the development, morphology and reproductive fitness of females and males in the sexually cannibalistic praying mantid *Pseudomantis albofimbriata*. Specifically, I was focused on (1) the effect of juvenile and adult feeding regime on the allocation of resources to female development and morphology, (2) the effect of juvenile feeding regime on the allocation of resources to male development and morphology (including the functionally significant antennae and wings) (3), the effect of juvenile and adult feeding regime on female reproductive fitness traits, and (4) the effect of juvenile feeding regime on male reproductive fitness traits. Males of this species are intense scramble competitors [23], so selection should favour those males that can quickly detect and mate with the best quality females [22]. Since previous studies of *P. albofimbriata* suggest that adult male size - which generally infers juvenile feeding - has little effect on the frequency of cannibalism [20], male mating success [20], proportion of paternity [23] or absolute sperm number transferred [25], I used only male mate location and mate choice ability as indictors of male reproductive fitness in this study. For females, I used fecundity and chemical attractiveness as indicators of reproductive fitness.

## Materials and Methods

### General methods

#### Collection and housing

Individual *Pseudomantis albofimbriata* were collected from various sites around Sydney, Australia, from January - February 2007 and 2009. The majority of individuals were found in *Lomandra longifolia* bushes at Kuringai Bicentennial Park and Yamba Reserve in Sydney, Australia. Juvenile animals were collected from the study sites and housed individually within well-ventilated 425 mL transparent cups in the laboratory, at a temperature of 24-26 °C and a diurnal period of 14 light hours per day. 

#### Measuring and sexing mantids

The pronotum length of all laboratory-reared mantids was recorded after the final moult and was used as a measure of fixed adult size, while body mass was measured immediately preceding mate choice experiments. I used body mass divided by fixed size *and* the residuals of a regression of body mass over fixed size as indices of body condition [28], however, both gave very similar results, so I report only fixed size divided by body mass throughout this paper. The sex of *P. albofimbriata* individuals was determined by differences in the adult abdomen and wing morphology.

#### Feeding regimes

After the collection in 2007, juvenile mantids (males n = 36, females n = 49) were placed into one of two feeding treatments: ‘L’ (low quantity) or ‘H’ (high quantity). So as to maintain some consistency with respect to the amount of time spent on feeding treatments, both males and females were placed into the treatment groups after attaining their penultimate instar [29]. However, twelve individuals were collected from the field during the penultimate instar, making it possible that they were on feeding treatments for less time than the other individuals (final instar duration, males = 19.857 d, females = 23.071 d; K Barry unpublished data). I allocated these particular individuals uniformly across treatment groups so as not to bias the results. The ‘normal’ feeding regime used in previous studies of this species is two small crickets three times per week [20,29], so individuals placed on the low-quantity feeding treatment in the current study were given one small cricket (mean cricket body mass = 0.037 ± 0.003 g, n = 50) three times per week, and individuals on the high-quantity treatment were fed three small crickets three times per week. Half of the females from the H treatment and half from the L treatment were then placed onto a high-quantity feeding regime as adults, and the other half were placed on a low-quantity feeding regime as adults. Female feeding treatments were renamed as follows: (1) ‘HH’ treatment (high quantity as a juvenile and high quantity as an adult, n = 13), (2) ‘HL’ treatment (high quantity as a juvenile and low quantity as an adult, n = 14), (3) ‘LH’ (low quantity as a juvenile and high quantity as an adult, n = 12), and (4) ‘LL’ (low quantity as a juvenile and low quantity as an adult, n = 10). Cricket remains were checked prior to subsequent feeding events to be sure that most food was being consumed. All adult males were placed on the intermediate feeding regime of two small crickets three times per week because previous studies showed that adult males were unlikely to consume more than this quantity (KL Barry unpublished). Therefore, only two male feeding treatments were used – the (1) ‘H’ treatment (high quantity as a juvenile and intermediate as an adult, n = 16) and the (2) ‘L’ treatment (low quantity as a juvenile and intermediate as an adult, n = 20) – and only the effect of juvenile nutrition on male development, morphology and reproductive fitness was measured. 

 The fixed size and body condition of all females raised in the laboratory was within the range of female sizes and conditions previously recorded in nature (range [fixed size] = 13.19 - 19.13 mm, n = 42; range [body condition] = 0.016 - 0.072, n = 42), and the fixed size and body condition of males raised in the laboratory was similar to the range of male sizes and conditions previously recorded in nature (range [fixed size] = 11.830 - 15.160 mm, n = 27; range [body condition] = 0.015 - 0.022, n = 27). 

#### Data analysis

Data were analysed using SPSS 20.0 for Mac and were checked for normal distribution (Kolmogorov–Smirnov test) before further statistical analysis. Unless otherwise stated, all values are mean ± standard error, and all statistical tests are two-tailed. 

#### Ethics statement

No permits were obtained for the described field collections/studies because New South Wales state law does not require specific permissions for the collection of invertebrates from locations outside of a national park. The field studies did not involve endangered or protected species.

### Aims 1 & 2: Effect of feeding regime on female & male development and morphology

The pronotum length, antennal length and wing length of individuals was measured using electronic callipers, and mass was measured using electronic scales. I used a two-way ANOVA with juvenile and adult feeding treatment as factors to compare the development time (number of days to reach adulthood), fixed size (pronotum length) and body condition (mass/size) of females. I used a one-way ANOVA to compare development time, fixed size, body condition, antennal length and wing length of males. The ratio of male antennal length/body size and wing length/body size was also related using an ANOVA. Wing length was not compared between female treatments because female wings are not functional in *P. albofimbriata*, and antennal length was not compared because, for reasons unknown, the distal ends of female antennae were often damaged and substantially shortened in length. Linear regression analyses were used to relate male morphological traits and to compare the resulting slope to b = 1.

### Aim 3: Effect of feeding regime on female reproductive fitness traits

#### Female fecundity

Female fecundity was measured as the total number of unfertilised mature eggs produced throughout a virgin female’s lifetime, and a two-way ANOVA was used to compare this measure between treatments (juvenile and adult feeding treatment as factors). 

#### Female attractiveness

I used a glass Y-maze olfactometer to measure female attractiveness, which allows males to make an active choice between two different chemical stimuli. These experiments were carried out during the early mornings from 27 March - 16 April 2007, as this is the most likely time for female pheromone emission in *P. albofimbriata* [19]. There were six combinations used during the experiment: choice between HH & HL female, HH & LH, HH & LL, HL & LH, HL & LL and LH & LL. Each male (n = 36) was given the opportunity to make two separate choices between two different and randomly allocated female combinations (each female used 2-3 times), however, two males did not make a definitive choice during the second test (first test n = 36, second test n = 34). Prior to each choice test, two females (each from a different treatment) were randomly allocated to a Perspex box and boxes were randomly allocated to the left or right position. The glass Y-maze tubes had a diameter of 2.3 cm and a length of 17 cm. Males were subsequently placed at the bottom of the maze and an air pump connected to the rear of each Perspex box via plastic tubing allowed airﬂow to be directed towards the male. Air was pumped past both boxes for approximately 1 min prior to the addition of the male so that any air-borne pheromones would be detectable. The anterior surface of each box was covered with an opaque cloth so that males could not use visual cues when making a choice. Males were given 1 h to move within the Y-maze and a response was recorded when they moved to the end of one of the Y-maze arms. Between each individual experiment, both Perspex boxes and the Y-maze tubing were washed with 95% ethanol so that the previous male and female scents did not affect the result of subsequent choices. To determine whether female attractiveness varied as a result of female feeding regime, I compared the total number of times (n = 70) a male was attracted to each of the four female treatments (as well as separate pairwise analyses) using a G-test. The size of fixed effects was estimated as the absolute difference between the number of ‘correct’ choices (i.e. number of trials in which the more fecund female was chosen) as a proportion of the total number of choices.

### Aim 4: Effect of feeding regime on male reproductive fitness traits

#### Mate location ability

I carried out mate location experiments in two large field enclosures (6 × 4 × 3 m) on the Macquarie University campus, North Ryde, Sydney in March - April 2007. These experiments were carried out to determine whether feeding condition affected the scrambling mate location behaviour known for *P. albofimbriata* males. Five small cages (30 × 20 cm diameter) – four containing virgin females in good condition (HH treatment) and one empty control cage – were placed in a random order around the interior perimeter of each enclosure, and five adult males arbitrarily chosen from the laboratory population were released onto foliage in the centre of each enclosure. All of the small cages housing the females were covered in two layers of garden mesh to obscure visual cues whilst still allowing any chemical signals produced by the females to escape. The cages were checked for males three times a day (7am, 2pm, 10pm) over a three-day period, and any male found on a cage was counted and immediately removed from the field enclosure. This experiment was repeated a second time with different males and females during the following week, and between each experiment the cages were washed with 95% ethanol so that the previous male and female scents did not affect the result of subsequent choices. Ten males from each of the feeding treatments (H and L) were used across the four enclosure trials, and data were subsequently pooled (n = 20 males in total) because there was no significant difference between enclosures (ANOVA p > 0.05). The frequency (z-test for proportions) and latency (t-test) to locate a female were compared between H males and L males. 

I also carried out supplementary mate location experiments in an unenclosed area of the Macquarie University campus, North Ryde, Sydney, from 23 - 28 February 2009. Unlike the feeding treatments in 2007, all males were maintained on the same diet of two small crickets three times per week and water daily, and all females received three small crickets three times per week and water daily. This experiment was carried out in addition to the field enclosure experiments so that the natural variation in male morphological traits could be correlated with male mate location ability in a natural setting. Six small mesh cages containing virgin females in good nutritional condition were placed haphazardly around the field site at least 20m apart, and twelve males from the laboratory population were released at least 10m apart and at least 10m from every female. Female cages were checked for incoming males three times a day (7am, 2pm, 10pm) over a three-day period, and the experiment was repeated a second time with new males and females in the following week (n = 24 males released in total). Wild males recaptured on females’ cages were removed and added to the laboratory population for subsequent use in unrelated studies. The fixed size and body condition of males were compared between those that successfully located a female and those that did not (t-test), and these measures were also correlated with the latency to locate a female (for those males that were successful). 

#### Mate choice ability

To determine whether male feeding regime had an effect on the ability of males to choose the best quality female, I used the data collected from the Y-maze olfactometer experiment carried out from 27 March - 16 April 2007 (see Aim 3 for more detailed methodology). First, I tested for an order effect between 1^st^ and 2^nd^ choice tests, and found there was no significant difference in the number of H and L males that chose the most fecund female during the 1^st^ round (H = 13/16, L = 18/20) and 2^nd^ round (H = 12/14, L = 19/20) of choice tests (Fisher’s exact test: p = 1). Therefore the choice data were pooled (n = 70), and a z-test for proportions was used to compare the total number of times H and L males chose the most fecund female.

## Results

### Aim 1: Effect of feeding regime on female development and morphology

Juvenile feeding affected development time and fixed female size so that females on the high treatments (HH + HL) became adults sooner and were of larger fixed size (see Table 1). Adult feeding, but not juvenile feeding, affected body condition so that females on the high treatments (HH + LH) were in better condition than females on the low treatments (Table 1). There was no significant interaction between the two factors for any of the traits measured. 

**Table 1:** A comparison of traits between individuals placed on different feeding regimes as juveniles (males & females) and adults (females).

	***JUVENILEFEEDING***		***ADULTFEEDING***			
	***High***	***Low***	***High***	***Low***	***Factor***	***Statistics***
***FEMALES***						
**Development time (days)**	21.225 ± 1.979	30.475 ± 2.200	23.529 ± 2.057	28.171 ± 2.127	*Juvenile feeding*	F_1,45_ = 9.770, **p = 0.003**
**Fixed size (mm)**	16.355 ± 0.198	14.895 ± 0.212	15.727 ± 0.198	15.523 ± 0.205	*Juvenile feeding*	F_1,45_ = 26.316, **p < 0.001**
**Body condition (mass/size)**	0.041 ± 0.002	0.038 ± 0.002	0.055 ± 0.002	0.024 ± 0.002	*Juvenile feeding*	F_1,39_ = 1.174, p = 0.285
					*Adult feeding*	F_1,39_ = 174.778, **p < 0.001**
					*Interaction*	F_1,39_ = 0.345, p = 0.560
***MALES***						
**Development time (days)**	18.833 ± 1.858	23.350 ± 1.435			*Juvenile feeding*	F_30_ = 3.707, p = 0.064*
**Fixed size (mm)**	13.749 ± 0.126	13.173 ± 0.198			*Juvenile feeding*	F_30_ = 4.394, **p = 0.045**
**Body condition (mass/size)**	0.018 ± 0.001	0.016 ± 0.001			*Juvenile feeding*	F_30_ = 6.170, **p = 0.019**
**Antennal length (mm)**	30.897 ± 0.800	29.084 ± 0.537			*Juvenile feeding*	F_24_ = 3.729, p = 0.065*
**Wing length (mm)**	29.721 ± 0.411	28.439 ± 0.415			*Juvenile feeding*	F_26_ = 4.040, **p = 0.050**
**Antennal length/fixed size**	2.237 ± 0.057	2.225 ± 0.030			*Juvenile feeding*	F_24_ = 0.043, p = 0.837
**Wing length/fixed size**	2.160 ± 0.035	2.177 ± 0.021			*Juvenile feeding*	F_26_ = 0.188 p = 0.668

Female F-values derive from two-way ANOVAs using high and low juvenile and adult feeding treatments as factors, and male F-values derive from one-way ANOVAs using high and low juvenile feeding treatments as factors. Significant p-values are highlighted in bold, and those approaching significance are marked with an asterisk.

### Aim 2: Effect of feeding regime on male development and morphology

There was a significant difference between the H and L treatments in fixed male size, body condition and wing size, and the difference in development time and antennal length approached significance (see Table 1). That is, males on the H treatment matured earlier, were larger in size, in better condition, and had longer antennae and wings than males on the L treatment. Although the difference between treatment groups for development and antennal length only approached significance, the effects are still noteworthy since males were only on feeding treatments for 2-3 weeks. Differences in antennal length and wing length disappeared when each was corrected for fixed size (Table 1). 

When comparing the variance (SE) as a percentage of the mean between male traits in the H treatment, I found that antennal length (24.7%) and wing length (12.2%) had much greater variation than fixed male size (1.7%). And although antennal length still had the greatest percentage variance for traits in the L treatment (antennal length = 15.6%; wing length = 11.8%; fixed male size = 2.6%), the difference was not as pronounced.

**Figure 1:**
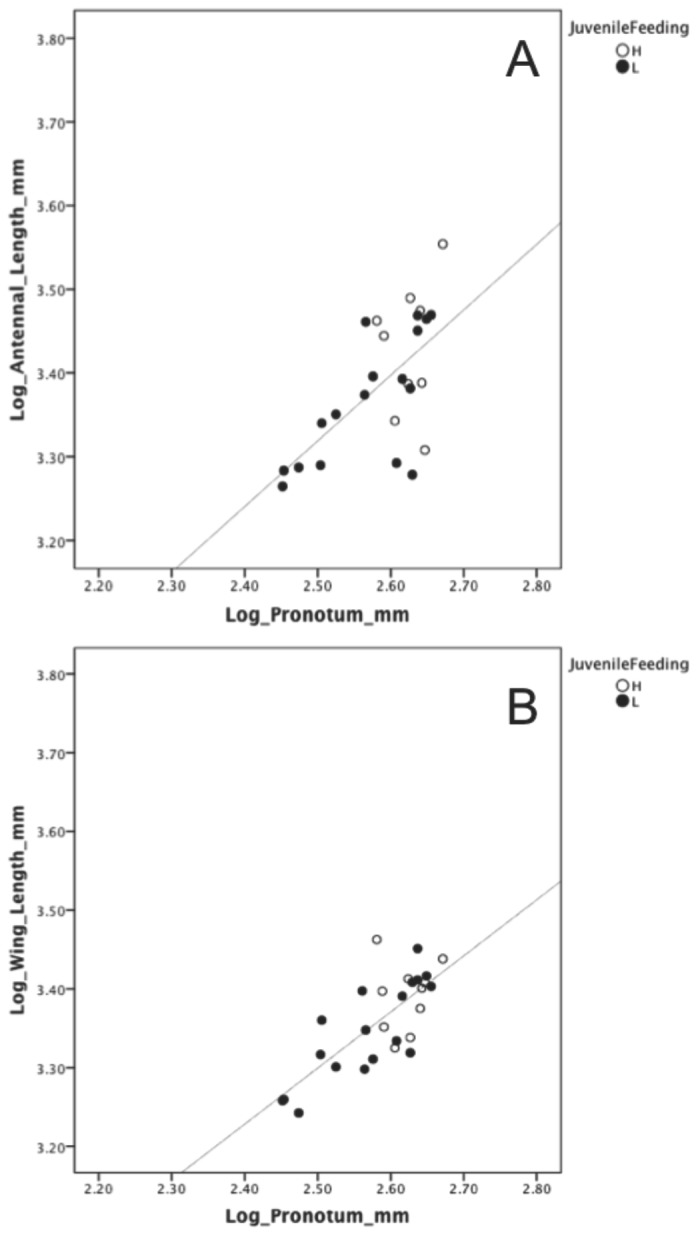
Male body size and antennal length/wing length. Scattergraphs showing the relationship between male body size and antennal length (A), and male body size and wing length (B). Body size was a significant predictor of antennal and wing length for low feeding treatment males (slope = 0.7432 and 0.7313, respectively), but not for high feeding treatment males.

Male body size significantly predicted antennal length (linear regression: b = 0.743, r^2^ = 0.480, F_1,16_ = 13.853, p = 0.002; Figure 1A) and wing length (linear regression: b = 0.731, r^2^ = 0.655, F_1,17_ = 30.335, p < 0.001; Figure 1B) for L males but not for H males (linear regression: [antennal length] r^2^ = 0.027, F_1,8_ = 0.196, p = 0.671; [wing length] r^2^ = 0.019, F_1,9_ = 0.157, p = 0.702). For L males, neither regression slope significantly differed from b = 1, however, the slope of body size/wing length approached significance (antennal length p = 0.1935; wing length p = 0.0639).

### Aim 3: Effect of feeding regime on female reproductive fitness traits

#### Female fecundity

**Figure 2:**
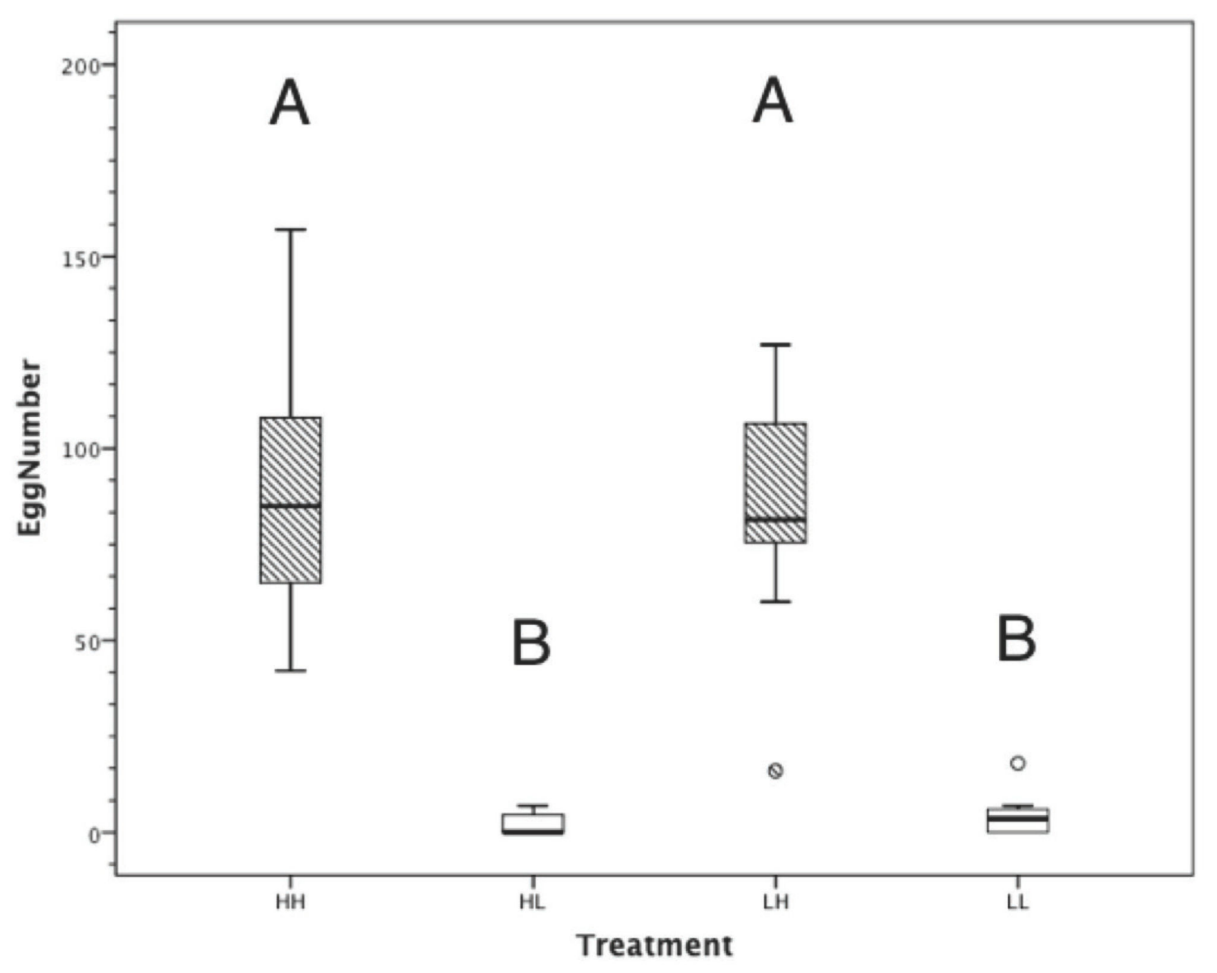
Feeding treatment and female fecundity. The females on high adult feeding treatments (A, n = 25) produced significantly more eggs than the females on low adult feeding treatments (B, n = 24), however, juvenile feeding treatment had no effect on fecundity. The horizontal line of box-plots represents the mean value for fecundity, the upper and lower boundaries of the box are the 75th and 25th percentiles and the bars represent the 90^th^ and 10th percentiles.

Adult feeding, but not juvenile feeding, affected fecundity so that females on the high treatments were more fecund than females on the low treatments (Table 2; Figure 2). There was no significant interaction between the two factors. Furthermore, regression analyses showed that female body condition was responsible for 72.4% of fecundity variation (r^2^ = 0.724, F_1,40_ = 104.795, p < 0.001), but only 0.2% of variation was explained by fixed female size (r^2^ = 0.002, F_1,45_ = 0.073, p = 0.788).

**Table 2:** A comparison of fitness measures between individuals placed on different feeding treatments as juveniles (males & females) and adults (females).

	***Factor***	***Statistics***	***Effect Size***
***FEMALES***			
**Fecundity**	*Juvenile feeding*	ANOVA: F_1,43_ = 0.000, p = 0.990	
	*Adult feeding*	ANOVA: F_1,43_= 142.526, **p < 0.001**	
	*Interaction*	ANOVA: F_1,43_ = 0.096, p = 0.758	
**Attractiveness**	*Total*	G-test: G_3_ = 24.935, **p < 0.001**	
	*Juvenile feeding*	G-test: G_1_ = 6.305, **p = 0.012**	0.286
	*Adult feeding*	G-test: G_1_ = 20.266, **p < 0.001**	0.516
	*HH vs HL*	G-test: G_1_ = 8.993, **p = 0.003**	0.588
	*HH vs LH*	G-test: G_1_ = 2.300, p = 0.129	0.358
	*HH vs LL*	G-test: G_1_ = 25.945, **p < 0.001**	0.802
	*HL vs LL*	G-test: G_1_ = 5.014, **p = 0.025**	0.214
	*LH vs LL*	G-test: G_1_ = 12.674, **p < 0.001**	0.444
	*HL vs LH*	G-test: G_1_ = 2.486, p = 0.115	0.230
***MALES***			
**Mate location success**	*Juvenile feeding*	z-test for proportions: z = 2.060, **p = 0.039**	
**Mate location latency**	*Juvenile feeding*	t-test: t_4.064_ = 1.331, p = 0.253	
**Mate choice ability**	*Juvenile feeding*	z-test for proportions: z = 1.131, p = 0.258	

Female F-values derive from two-way ANOVAs using high and low juvenile and adult feeding treatments as factors. Significant p-values are highlighted in bold and effect sizes are included.

#### Female attractiveness

**Figure 3:**
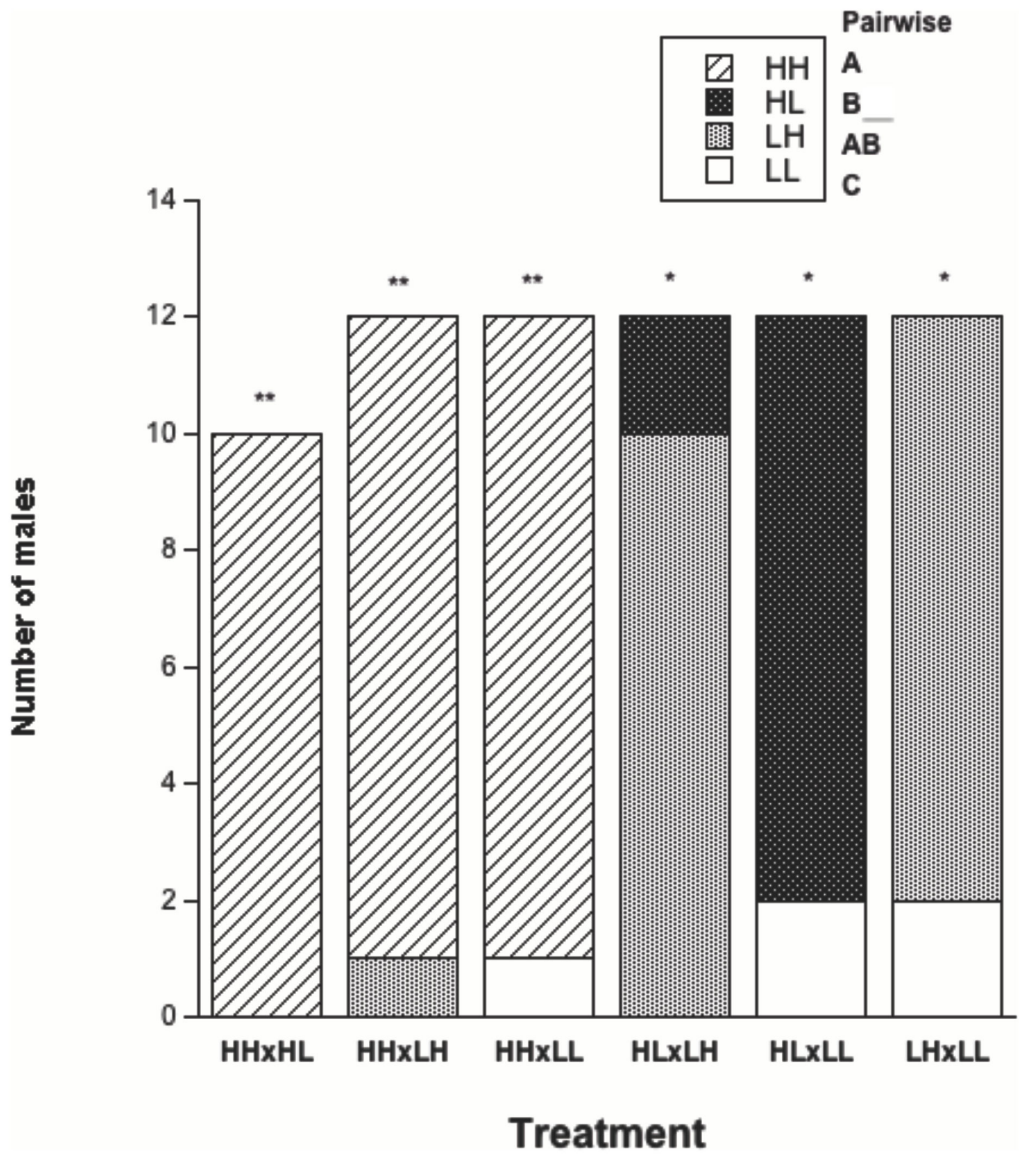
Feeding treatment and female attractiveness. There was a significant difference (p < 0.05*, p < 0.01**) in attractiveness between the four groups (HH = 32, HL = 12, LH = 21, LL = 5), with the results of pairwise analyses depicted by letters (A, B, C) in the top right of the graph.

There was a significant effect on female attractiveness via airborne pheromone production as a result of overall feeding treatment (Table 2; Figure 3). Further pairwise analysis showed a significant difference between 4/6 comparisons (Table 2). There was no significant difference in the total number of males attracted to HH/LH and HL/LH females, and effect sizes were also relatively small for these two pairwise comparisons. However, HH females were more attractive when in direct competition with LH females (Binomial test: k = 10, n = 11, p = 0.012) and LH females were more attractive when in direct competition with HL females (Binomial test: k = 10, n = 12, p = 0.039). Food limitation during the juvenile stages had a negative effect on attractiveness irrespective of adult feeding, and food-limitation during adulthood had an even more pronounced negative effect irrespective of juvenile feeding (Table 2). Total effect size was larger for adult feeding than for juvenile feeding, and effect sizes were also larger for adult feeding than for juvenile feeding in the pairwise comparisons (Table 2). The largest effect occurred between low adult and low juvenile feeding (LL) versus high adult and high juvenile feeding (HH).

### Aim 4: Effect of feeding regime on male reproductive fitness traits

#### Mate location ability

For field enclosure experiments, H males were more successful at finding females than L males: 9 out 10 males (90%) from the H treatment and 5 out of 10 males (50%) from the L treatment (Table 2). When they were successful, H males were also faster to find females (15.600 ± 0.600 h) than L males (24.600 ± 6.735 h; Table 2), however the difference was not statistically significant. 

In the supplementary field experiment where male feeding was unmanipulated, there was no significant difference in the body condition or fixed size of males that successfully located a female (fixed size = 13.698 ± 0.253 mm, body condition = 0.018 ± 0.001, n = 6) and those males that were unsuccessful (fixed size = 13.591 ± 0.166 mm, body condition = 0.018 ± 0.001, n = 18; t-test [fixed size]: t_22_ = 0.329, p = 0.745; t-test [body condition]: t_22_ = 0.372, p = 0.713). There was, however, a significant negative correlation between the latency to locate a female and male fixed size (Pearson’s: r = -0.933, n = 6, p = 0.007) and male body condition (Pearson’s: r = -0.828, n = 6, p = 0.042): larger males that were in good condition were quicker to locate a female.

#### Mate choice ability

There was no significant difference in the ability of males on different feeding regimes to choose the fittest female (Table 2): the most fecund female was chosen in 25 out of 30 trials (83.3%) undertaken by H males and in 37 out of 40 trials (92.5%) undertaken by L males. It is also worth noting that no male chose the less fecund female during both choice opportunities (irrespective of feeding treatment).

## Discussion

### Effect of feeding regime on development and morphology

There was a trend for males and females that were food-limited as juveniles to develop more slowly, achieve a smaller fixed size and shorter antennae and wings (males only) than food-satiated individuals. Effects such as these are common for insects in general [1-3,30,31], and for praying mantids more specifically [15,17]. Substantial changes to the developmental schedule are expected to have a considerable impact on females in nature, particularly for univoltine individuals living in temperate regions. First, late-maturing females are unlikely to have enough time to produce eggs, mate and lay an egg case (estimated at ~4 weeks minimum for *P. albofimbriata*)[20] before the cool temperatures of autumn and winter set in. Second, slower development may mean a reduced potential for female mate choice/opportunity because sexual cannibalism is thought to skew the sex ratio towards females later in the mating season [32]. The effects of late development may not be as pertinent for males as for females because males do not have the reproductive time constraints associated with oogenesis and oviposition, however, delayed maturity might still have a significant effect, most notably on the ability to locate a female before other males [33,34] or on the number of virgin females available to mate with later in the season. In *P. albofimbriata*, mated females are no longer chemically attractive to males [23], so a lack of virgin females could mean a substantial decrease in reproductive success for food-limited males in nature. 

 With respect to the allocation of resources to different body parts, there was a significant difference/trend in all traits between H and L males and no significant difference in trait ratios (i.e. antennal length/body size and wing length/body size). This suggests a proportional distribution of resources among male traits and shows no evidence of any trait conservation by food-limited males, as has been shown for other animals [13,14,35]. Furthermore, male body size significantly predicted antennal and wing length in only food-limited males. That is, L males with the smallest body sizes also had the shortest wings and antennae and larger body size meant longer wings and antennae. Both antennal length and wing length showed an isometric relationship with respect to body size in the food-limited treatment, and this kind of relationship is usually thought to characterise non-sexually selected traits [36,37]. Thus the finding of an isometric relationship for traits considered to be under sexual selection in this species [22,23] contradicts the traditional views on the effect of sexual selection on trait allometry. However, a recent review of the literature suggests the widely held view that almost all sexually selected traits are positively allometric is inconsistent with empirical evidence and theory [38]. House and Simmons [39] further suggest that the amount of variation in traits under directional sexual selection may be limited by natural selection, which might explain the absence of positive allometry in a trait that is related to locomotion or other viability related functions. In *P. albofimbriata*, natural selection may constrain the size of wings because oversized wings might have a negative effect on manoeuvrability and speed during flight. In regards to the isometric antennae, it may be that the length of antennae is constrained by natural selection, and the trait under sexual selection is absolute number or density of antennal sensilla. Conversely, there was no obvious relationship between morphological traits in H males, which suggests that these males are strategically allocating the extra resources obtained from a high-quantity diet to a particularly important trait. In rhinoceros auklet chicks, individuals fed on small meals maintained the growth of organs essential for fledging, and those fed on large meals deposited the surplus as lipid rather than allocating more to the development of organs [35]. In the current study of *P. albofimbriata*, there was relatively little variation in male body size compared to variation in the other two traits, potentially indicating that males with an excess of resources strategically allocate an optimal quantity to body size before allocating to the other traits. 

### Effect of feeding regime on female reproductive fitness traits

Female fecundity was not significantly affected by food limitation during the juvenile stage, however it was strongly affected by adult feeding treatment. With respect to juvenile feeding, LH females produced the same number of eggs (on average) as the HH females, even though HH females were of substantially larger fixed size. In addition, only 0.2% of variation in fecundity is explained by fixed size. These results suggest that poor juvenile feeding can be compensated for during adulthood in *P. albofimbriata*, directly contradicting the results from studies of two closely-related species [9,15]. With respect to adult feeding treatment, the mean body condition of females in the high treatments was more than twice that of females in the low treatments, and this translated to a 20-fold increase in the number of eggs produced. There are many examples of this effect throughout the insect world [40], and more specifically in praying mantids [15,18,19]. These results further explain why food-limited praying mantids use sexual cannibalism as a foraging strategy - to increase their fecundity [18,20,21,41,42]. It should, however, be noted that any boost in female fecundity as a result of sexual cannibalism is unlikely to compensate for the level of adult food limitation imposed by the experimental treatments in the current study. Using the regression equation from Barry et al. [20], an average food-limited female from the current study would increase her body condition by approximately 1 unit as a result of consuming a male, which translates to a 3-fold increase in fecundity (from 3 eggs to 9 eggs). Although this is a significant increase relatively speaking, it does not offset consistent adult food limitation (~80 eggs for HH and LH females).

 Female attractiveness was significantly affected by overall treatment group, creating a sliding scale of attractiveness most notably affected by adult feeding regime (HH = 32/34 (94.1%) > LH = 21/36 (58.3%) > HL = 12/34 (35.3%) > LL = 5/36 (13.9%)). That adult diet significantly affected female attractiveness is not unexpected: both body condition and fecundity were much higher in the food-satiated females than in the food-limited females. Diet and body size/condition have been shown to affect pheromone production and subsequent attractiveness in a variety of species [43-45], including praying mantids [18,19,27,46,47]. Severe and consistent food limitation during adulthood may totally constrain egg production, which has the flow-on effect of complete chemical unattractiveness in *P. albofimbriata* [19], making mating highly improbable for these females in nature. The more likely scenario though, is that food limitation is not so extreme and therefore results in at least a few eggs being produced over time. In *P. albofimbriata*, 1-2 eggs in the ovaries is enough to make a female chemically attractive to males [19]. However, being attracted to one of these females means far fewer eggs to fertilise *and* a greatly increased risk of sexual cannibalism [20], making this a considerable cost for males and a potential sexual conflict in this species. That juvenile feeding affected chemical attractiveness seems counterintuitive at first: a previous study [19] showed that egg production is intimately linked to pheromone production in *P. albofimbriata*, and juvenile feeding had no effect on body condition or fecundity in the current study. However, when taking the experimental set-up into consideration, this result makes more sense. Each male makes a choice between two females from different treatments, and theory predicts he should choose the most fecund female. It may not be important that the average fecundity difference between treatments is statistically significant, only that the difference between individual females is enough for males to perceive during each independent choice. It should, however, be noted that the effect of juvenile feeding on attractiveness was only half as strong as the effect of adult feeding, that the pairwise effects were larger when the treatments differed in their adult feeding regime as opposed to their juvenile feeding regime, and that adult feeding had a greater effect than juvenile feeding on the outcome of individual choice tests when feeding regime contrastingly differed in both life history stages (i.e. HL vs LH). Most interestingly, the smallest effect size was generated for treatment groups with varied juvenile feeding and low adult feeding (i.e. HL vs LL) even though the level of attractiveness was statistically different. These results suggest that the effect of juvenile food limitation on female chemical attractiveness is quite complex and requires further investigation.

### Effect of feeding regime on male reproductive fitness traits

Juvenile feeding treatment had an effect on long distance mate location ability so that H males were more successful at finding females in field enclosure experiments. Although the difference in the latency to find a female was not statistically significant, the mean difference of nine hours is biologically significant because it is more than enough time for a male to mate (6 hours on average)[20] and render a female chemically unattractive to other males [23]. If he can achieve this before other males are initially attracted to the female, it is likely he will secure 100% paternity. There was also a negative correlation between size/condition and latency to find a female in the field experiments where food quantity was not manipulated. Bigger size and/or body condition as an adult infers more food as a juvenile, which provides further evidence that juvenile food limitation negatively affects long distance mate location ability in adult mantids. These results are in stark contrast to those predicted by scramble competition theory [48], which proposes that small males may have an indirect advantage if they mature earlier (and therefore smaller) or a direct advantage if small size means greater agility [34,49,50]. However, it has also been suggested that large size might mean higher survivorship during mate searching over the long distances expected when females are sparsely distributed [34,51-53]. The current study of *P. albofimbriata* provides some of the first empirical evidence for the latter prediction. Although food limitation had an effect on some aspect of long-distance male mate location ability in both the field enclosure and field site experiments, it did not have an effect on the ability of males to choose the fittest female in short-range simultaneous choice tests. It may be that food limitation and its subsequent effect on morphological traits only has a significant negative impact in the more ecologically relevant scenario of long distance mate location [27] and in the harsh and more variable conditions of nature.
